# Endothelial Dysfunction: Redox Imbalance, NLRP3 Inflammasome, and Inflammatory Responses in Cardiovascular Diseases

**DOI:** 10.3390/antiox14030256

**Published:** 2025-02-23

**Authors:** Claudia Penna, Pasquale Pagliaro

**Affiliations:** 1 Department of Clinical and Biological Sciences, University of Turin, 10043 Orbassano, Italy; claudia.penna@unito.it; 2 National Institute for Cardiovascular Research (INRC), 40126 Bologna, Italy

**Keywords:** nitric oxide, oxidative stress, NLRP3 inflammasome, antioxidant treatments, metabolic syndrome, ischemia/reperfusion injury

## Abstract

Endothelial dysfunction (ED) is characterized by an imbalance between vasodilatory and vasoconstrictive factors, leading to impaired vascular tone, thrombosis, and inflammation. These processes are critical in the development of cardiovascular diseases (CVDs) such as atherosclerosis, hypertension and ischemia/reperfusion injury (IRI). Reduced nitric oxide (NO) production and increased oxidative stress are key contributors to ED. Aging further exacerbates ED through mitochondrial dysfunction and increased oxidative/nitrosative stress, heightening CVD risk. Antioxidant systems like superoxide-dismutase (SOD), glutathione-peroxidase (GPx), and thioredoxin/thioredoxin-reductase (Trx/TXNRD) pathways protect against oxidative stress. However, their reduced activity promotes ED, atherosclerosis, and vulnerability to IRI. Metabolic syndrome, comprising insulin resistance, obesity, and hypertension, is often accompanied by ED. Specifically, hyperglycemia worsens endothelial damage by promoting oxidative stress and inflammation. Obesity leads to chronic inflammation and changes in perivascular adipose tissue, while hypertension is associated with an increase in oxidative stress. The NLRP3 inflammasome plays a significant role in ED, being triggered by factors such as reactive oxygen and nitrogen species, ischemia, and high glucose, which contribute to inflammation, endothelial injury, and exacerbation of IRI. Treatments, such as N-acetyl-L-cysteine, SGLT2 or NLRP3 inhibitors, show promise in improving endothelial function. Yet the complexity of ED suggests that multi-targeted therapies addressing oxidative stress, inflammation, and metabolic disturbances are essential for managing CVDs associated with metabolic syndrome.

## 1. Introduction

Despite its simple monolayer structure, healthy endothelium responds to a variety of stimuli, both chemical and mechanical. In turn, the endothelium is capable of producing a wide range of molecules that regulate vascular permeability, cell adhesion, smooth muscle cell proliferation, vessel wall inflammation, and thrombosis resistance.

Endothelial dysfunction (ED) is characterized by an impaired response of endothelial cells (ECs) to physiological stimuli, resulting in altered vascular tone and organ perfusion. It arises from an imbalance between vasodilator and vasoconstrictor factors and is associated with various conditions, including neurodegenerative diseases, kidney disease, metabolic syndrome, and cardiovascular diseases (CVDs) [1].

Sharing mechanisms with many of the aforementioned pathological conditions, ED often serves as a starting point, highlighting a common link among these disorders. This raises the question of whether early detection of ED could be valuable for the screening and prevention of CVDs.

From a clinical point of view, the evolution of ED can be followed by measuring some molecules in order to obtain an early diagnosis. Molecules identified as markers of endothelial dysfunction include cytokines such as interleukins (e.g., IL-6 and IL-1), tumor necrosis factor-alpha (TNF-α), adhesion molecules like intercellular adhesion molecule (ICAM) and vascular cell adhesion molecule (VCAM), as well as endothelial microparticles [2].

The molecular mechanisms underlying ED are numerous and not entirely clear, despite the fact that this condition has been discussed since the late 1970s [3]. Certainly, among the most accredited causes are disruption in nitric oxide (NO) production and the formation of reactive oxygen species (ROS) and/or nitrogen species (RNS). Yet hemodynamic and shear stress alterations, including turbulence, play a central role in ED development [4]. Indeed, triggers for the shift toward ED and, therefore, toward a pro-coagulant and pro-inflammatory state include intrinsic, non-modifiable risk factors, such as hemodynamic disturbances and a predisposition to thrombophilia (whether inherited or acquired). Modifiable risk factors also play a role, including infections, dietary disturbances, metabolic disorders such as homocysteinemia and hypercholesterolemia, and exposure to toxins like those from smoking [1,5,6].

The inflammatory response plays a central role by activating the NOD-like receptor family pyrin domain-containing 3 (NLRP3) inflammasome. This activation triggers the release of cytokines, which in turn fuel oxidative stress and further inflammatory processes, creating a vicious cycle [7,8,9].

In this brief review, ED pathology will be considered in terms of redox and inflammatory response, since both these factors are among the main causes of NLRP3 activation and CVDs associated with ED.

## 2. Features of Healthy Endothelium

Healthy ECs lining the inner wall of vessels form a dynamic interface with different functions on the surrounding microenvironment. Vascular homeostasis in healthy endothelium is maintained through the release of NO, produced mainly by endothelial NO synthase (eNOS). The endothelium controls the functions of the organs and their metabolism primarily in a paracrine manner through what is defined as “angiocrine signaling” [10]. Indeed, the endothelium regulates blood flow and vascular resistance by producing several factors, including vasodilators (e.g., NO and prostacyclin (PGI_2_)) and vasoconstrictors (e.g., endothelins (ETs) and thromboxane A_2_ (TxA_2_)), ensuring the appropriate delivery of oxygen and nutrients to tissues. Moreover, ECs play a crucial role in maintaining circulatory and blood homeostasis. In their normal state, ECs produce various substances, including prostaglandins, PGI_2_, carbon monoxide (CO), and adenosine diphosphatase, which modulate platelet activation and aggregation, maintaining vascular homeostasis [11,12,13,14,15,16,17,18,19,20,21,22,23] (Table 1). Endothelial cells also secrete anticoagulant proteins, such as thrombomodulin and heparin-like molecules, and promote fibrinolysis by synthesizing tissue plasminogen activator (t-PA). The activity of the endothelium is physiologically controlled and mediated not only by various factors, but also through a signaling involving mechanotransduction [4]. Indeed, besides the aforementioned factors, vascular homeostasis is also regulated by myoendothelial junctions (MEJs), which mediate direct interactions between ECs and vascular smooth muscle cells (VSMCs) in resistance vessels. MEJs facilitate endothelial-dependent hyperpolarization (EDH), a NO-independent mechanism modulating vasodilation and vasoconstriction. Recent studies have highlighted the role of membrane proteins such as Transient Receptor Potential Vanilloid 4 (TRPV4), Piezo1, Acid-Sensing Ion Channel 1a (ASIC1a), Connexins, and eNOS in vasomotor signal initiation and propagation. Although eNOS is not an intrinsic membrane protein, its localization to caveolae and interaction with caveolin-1 is promoted by post-translational modifications. Additional insights into Notch signaling have further elucidated its impact on vascular tone [4,12,13]. Initially identified as a vasodilator factor distinct from NO and PGI2, EDHF’s identity evolved into the broader concept of EDH signaling, reflecting its complex role in vascular function. EDH signaling involves hyperpolarization originating in ECs and transmitting to VSMCs via two pathways: chemical (e.g., potassium ions, lipid mediators, hydrogen peroxide (H_2_O_2_)) and electrical (via gap junctions, including MEJs). These pathways vary in contribution depending on arterial location, branch order, and VSMC activation. MEJs mediate bidirectional communication between ECs and VSMCs. This dynamic interaction enables feedback mechanisms like Ca^2+^ transfer during vasoconstriction, activating eNOS and EDH factor (EDHF) release to regulate vascular tone. MEJs also coordinate vasomotion, a cyclic process of vasodilation and constriction, which optimizes blood flow [14].

Beyond the functions mentioned earlier, ECs modulate the metabolism and transport of nutrients and hormones in an organ-specific manner, thereby serving as critical regulators of systemic metabolic homeostasis [23]. Moreover, ECs limit lipoprotein oxidation by reducing oxidative stress in the vascular wall through the production of NO, which is crucial in preventing atheromatous plaque formation [11,15,23]. The endothelial function is also regulated by the activation of receptor tyrosine kinases, such as vascular endothelial growth factor receptor (VEGFR2 and VEGFR3), as well as ion channels, integrins, and junctional proteins. Examples of junctional proteins are platelet endothelial cell adhesion molecule-1 (PECAM-1) and vascular endothelial (VE)-cadherin) [24].

Blood flow at the vessel level determines the onset of physical forces and mechano-transduction at the endothelial cell level. In physiological conditions, the shear stress produced by this force maintains the homeostasis of the endothelium. When this physical force varies—whether it increases or decreases—turbulence can arise, potentially disrupting homeostasis. Among the causes that can determine an alteration of shear stress, it is worth mentioning the increase in blood pressure which, combined with a greater stiffness due to metabolic syndrome and aging, determines changes in both the phenotype and the endothelial functionality [4,25].

In addition to being subjected to shear stress, ECs can function as a “metabolic interface” with important differences between different organs and within the organ itself [26,27]. It appears that under many conditions, the preferred metabolic pathway of ECs is anaerobic glycolysis [28]. The reliance on anaerobic glycolysis offers metabolic advantages. Under normal conditions, it reduces the formation and availability of ROS/RNS while enhancing the efficiency of metabolite transport from the vessel to surrounding tissues. For instance, the endothelium exerts metabolic control over surrounding tissues, regulating the influx of hormones and other key elements such as insulin, lipids, and glucose. Anaerobic glycolysis is also used during angiogenesis, thus favoring the invasion of less oxygenated areas by the endothelium [29].

*In summary*, ECs play a crucial role in maintaining vascular homeostasis through the release of NO, produced mainly by eNOS. The endothelium regulates blood flow, vascular resistance, and tissue metabolism via paracrine signaling, producing both vasodilators like NO, PGI_2_, and CO, and vasoconstrictors such as endothelins. Vascular homeostasis is also controlled by MEJs, which mediate communication between ECs and VSMCs, influencing vasodilation and vasoconstriction. EDH signaling, which involves hyperpolarization of VSMCs, is also vital in regulating vascular tone. Endothelial function is further influenced by mechanical forces such as shear stress, which maintains endothelial health. Additionally, ECs produce anticoagulant proteins and promote fibrinolysis. The endothelium also participates in metabolic processes like hormone regulation and lipoprotein oxidation, and it relies on anaerobic glycolysis for energy, particularly during angiogenesis. Due to its multiple functions and interactions, as well as the vastness of its surface, the endothelium plays a pivotal role in maintaining cardiovascular and overall organism health. Therefore, ED is at the foundation of a wide range of pathological conditions.

## 3. Endothelial Dysfunction and Activation

By definition, ED is a pathological state of the endothelium characterized by an imbalance between the production of vasodilatory substances and vasoconstrictive, pro-inflammatory, and pro-thrombotic factors. ED impairs the endothelium’s ability to regulate vascular tone, maintain vascular homeostasis, and prevent thrombosis and inflammation. ED contributes to the development and progression of CVDs, such as atherosclerosis and hypertension.

Endothelial activation is a state in which ECs upregulate the expression of adhesion molecules (e.g., VCAM-1, ICAM-1), pro-inflammatory cytokines, chemokines, and pro-coagulant factors in response to various stimuli, such as inflammatory signals, oxidative stress, or shear stress disturbances. This process facilitates leukocyte adhesion and transmigration, promotes inflammation, and contributes to vascular remodeling and thrombosis, playing a critical role in immune responses and CVDs. When the endothelium is damaged, its balance is disrupted, and the release of cytokines (e.g., ILs and TNF-α) contributes to pathological processes. Also, alterations in intracellular pathways contribute to pathological processes. For instance, studies have reported that reduced fatty acid transport in the heart, resulting from disruptions in the endothelial Notch signaling pathway, can lead to congestive heart failure [19,30,31]. In skeletal muscle following an alteration of the trans-epithelial transport of fatty acids, the onset of insulin resistance in obesity can be recorded [32]. In this context of dysfunction and activation, the endothelium is both a victim and culprit source and a target of oxidative stress.

**Figure 1:**
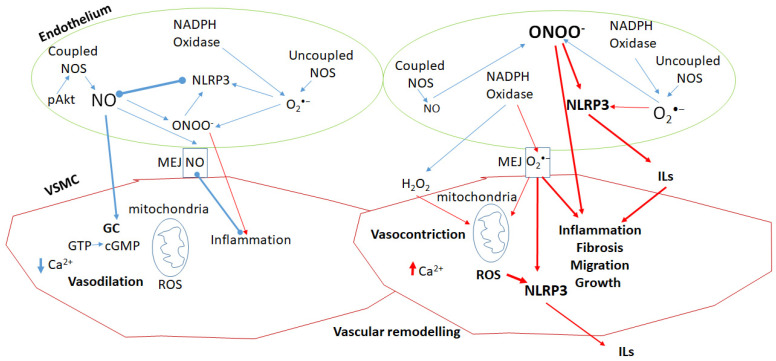
Adequate NO production supports redox balance, vasodilation, and vascular homeostasis. In contrast, endothelial dysfunction and reduced NO levels lead to redox imbalance, favoring ROS/RNS-mediated inflammation and fibrosis, with O_2_^•−^, ONOO^−^ and NLRP3 playing key roles (See also Figure 2). Double-headed lines indicate inhibition, while simple arrows indicate diffusion and activation. Red-colored arrows indicate a pathway linking oxidative stress to inflammation. Abbreviations are as in the text.

No doubt, a key substance produced by the healthy ECs is NO. Typically, the endothelium maintains its functional integrity and adequately responds to the needs of different organs when NO production and release are appropriately regulated. When the release of NO is lacking and/or the production is reduced, the vascular balance is disrupted and therefore ED occurs, which determines a pro-inflammatory and pro-thrombotic condition, together with a less compliant structure at the vascular level [33]. A co-protagonist is the increase in oxidative/nitrosative stress. The ROS/RNS increase could be caused on the one hand by a poor antioxidant defense and on the other by a greater production caused by vascular damage and specific pro-oxidant pathways [2,34,35,36]. It should be noted that NO possesses significant antioxidant properties, helping to neutralize ROS/RNS and maintain cellular redox balance (Figure 1). Indeed, the interactions between NO, ROS, and RNS form a dynamic redox system operating on a millisecond timescale to maintain homeostasis. A deficiency in NO (e.g., uncoupled NOS prevails) paradoxically increases both oxidative and nitrosative stress, showing a U-shaped relationship between NO levels and stress. While NO reacts with O_2_^•−^ to form ONOO^−^, it also scavenges this and other RNS, mitigating their damage. Inadequate NO impairs this protective mechanism [35,36,37,38,39,40] (Figure 1). (See also Section 3.1).

Elevated oxidative stress can further damage ECs, driving inflammation and accelerating the activation and progression of vascular diseases. This creates a vicious cycle in which reduced NO availability amplifies oxidative damage, aggravating ED and endothelial inflammation. Ultimately, this contributes to the development and progression of cardiovascular conditions such as atherosclerosis and hypertension, accompanied by cytokine release and adhesion molecule expression at the endothelial cell surface. Therefore, the inflammatory state of ECs plays a crucial role in the progression of CVDs. For instance, in metabolic syndrome and obesity, there is a chronic low-grade inflammatory state, which can determine ED, causing alterations in the endocrine and paracrine action of adipose-derived factors. In healthy subjects, perivascular adipose tissue promotes vasodilation, whereas in obese individuals, a significant change in adipocytokine release is observed, leading to a reduced vasorelaxant effect and progression toward CVDs [41]. Moreover, ED in CVDs disrupts EDH signaling, shifting vasomotion toward pathological vasospasm. This underscores the pivotal role of EDH signaling in maintaining vascular homeostasis and highlights its potential as a therapeutic target [14].

Of note, ED is “physiologically” induced by aging. In fact, with age, there are evident changes of both a structural and functional nature which contribute to the onset of CVDS diseases. At the cellular level, in addition to the classic signs of ED, trans-differentiation of VSMCs and cellular senescence have been observed. With aging, numerous changes at the molecular level of ECs are recognized. In larger vessels, such as the aorta, an increase in end-to-end inter-endothelial junctions is observed during aging, while overlapping or interdigitated junctions decrease [42]. The carotid arteries exhibit increased permeability in the elderly due to the separation of endothelial cell–cell junctions [43]. In several vessels, including the aorta and coronary arteries, both an increase in endothelial apoptosis caused by aging and a reduced cell density are found [42,43]. Increased autophagy has been reported to reverse arterial aging by improving age-related ED [43,44,45]. Aging is also among the factors that determine mitochondrial dysfunction. In fact, vascular ECs present impaired mitochondrial biogenesis, reduced respiration, and increased production of ROS, which directly contribute to a reduced NO availability [46]. Oxidative stress can also disrupt mitophagy in vascular cells by impairing the PTEN-induced kinase 1 (PINK1)/Parkin pathway and reducing the expression of key mitophagy receptors, including BCL2 interacting protein 3 (BNIP3) and Nox [47,48,49]. This disruption leads to the accumulation of dysfunctional mitochondria, contributing to ED, impaired angiogenic response to injury, and cardiac remodeling [50]. Physiological aging induces phenotypic changes that make the vascular system more fragile in its various components, thereby increasing susceptibility to CVDs, such as hypertension, cardiac remodeling, and other pathologies related to metabolic syndrome [43] (See also Section 4).

### 3.1. Focus on Oxidative and Nitrosative Stress

Oxidative and nitrosative stresses are a disturbance in the redox state of the cell, in which the production of ROS and RNS overcomes the antioxidant defenses. RNS are highly reactive molecules derived from NO. Examples of RNS include ONOO^−^, nitrogen dioxide (NO_2_), dinitrogen trioxide (N_2_O_3_), nitroxyl anion (HNO/NO^−^), and S-nitrosothiols (RSNOs) [11,15,35,39,40]. These molecules can participate in both physiological signaling and pathological processes, depending on their concentration and cellular context. In particular, in the case of oxidative stress due to RNS, the alteration of NO signaling is observed due to a relative increase in the amounts of RNS or by an inappropriate production, which can be caused by or associated with a disturbance in the redox state [35,36,37,38,39,40]. Oxidative and nitrosative stresses contribute to a cascade of events that drive both cell death and the increased expression of various inflammatory mediators. These stresses at the endothelial level can be caused by various factors, such as oxidized low-density lipoproteins (oxLDLs), aging, and hyperglycemia. The production of ROS primarily derives from dysfunctional mitochondria, uncoupling of eNOS, xanthine oxidase, and NADPH oxidase (NOX) [51,52]. In several conditions, eNOS undergoes uncoupling (See below), leading to the production of superoxide anion (O_2_^•−^) and the subsequent formation of ONOO^−^. The generated ROS and RNS further diminish the availability of tetrahydrobiopterin (BH_4_), impairing NO production by ECs [53].

In physiological conditions, vascular and endothelial NOX enzymes have a very limited role [54], while in pathological conditions in response to acute or chronic stimuli, their activity increases significantly, compromising vascular functionality [55]. The isoforms present at the vascular level are NOX1, NOX2, NOX4, and NOX5. The main isoform at the endothelial level is NOX4, which is responsible for the production of H_2_O_2_, unlike the other isoforms, specialized in the synthesis of O_2_^•−^ [54]. The role of individual NOX forms has long been studied and some isoforms are associated with ED. For example, NOX2 increased activity has been observed in various conditions, including dyslipidemia, obesity, smoking, hypertension, and aging [55,56]. In apolipoprotein E knockout (ApoE^−/−^) mouse models, NOX2 levels were significantly increased, and these animals also exhibited endothelial activation [55,56]. In mouse models, endothelial-specific overexpression of NOX2, following treatment with angiotensin II, a greater production of ROS, and a reduced response to acetylcholine were measured compared to the control mouse. The induced ED was associated with increased activity of NOX2, which contributed to vascular remodeling and hypertension [57]. The role played by other NOX isoforms is not very clear yet. For instance, NOX4 plays a significant role in oxidative stress and neuronal injury, making it a potential therapeutic target for stroke and related conditions. Indeed, in NOX4 knockout (NOX4^−/−^) mouse models, where other NOX isoforms remained, the absence of NOX4 conferred protection against oxidative stress and was associated with reduced neuronal apoptosis following stroke, highlighting NOX4′s role in neuronal damage under oxidative stress conditions [54,58]. Similar trends were observed with cardiac Nox4 deletion, where pressure overload-induced manifestations, including cardiac hypertrophy, fibrosis, and apoptosis, were significantly reduced [54,59].

Moreover, when ROS overwhelm NO production, increased tyrosine nitration occurs, indicating elevated ONOO^−^ production, which can lead to vascular damage [60]. Excess ROS also inhibits glyceraldehyde 3-phosphate dehydrogenase (GAPDH), resulting in increased diacylglycerol levels and activation of protein kinase C (PKC), which further stimulates ROS production through NOX activation [61].

There are also additional stimuli capable of increasing the production of ROS in ECs; for instance, ischemia/reperfusion exposes ECs to stimuli that amplify ROS production, driving endothelial dysfunction. Ischemia upregulates sodium-hydrogen exchangers (NHE), increasing intracellular Na^+^ and cytosolic Ca^2+^ via the sodium-calcium exchanger (NCX) [62]. Elevated Ca^2+^ activates the PKC-NOX pathway, thereby further increasing ROS [63]. Excess ROS oxidize lipids and proteins while impairing antioxidant enzymes like catalase and SOD. The oxidative stress activates the vascular NLRP3 inflammasome, exacerbating endothelial dysfunction, the no-reflow phenomenon, and tissue damage during reperfusion [64,65,66].

Following senescence at the level of ECs, an increase in the expression of pro-inflammatory cytokines is observed, thus hypothesizing that aging is a cause of the compromised of vascular tone caused by the accumulation of nitro-oxidative stress and mitochondrial damage [67].

*In summary*, ED occurs when the balance between vasodilatory and vasoconstrictive factors is disrupted, leading to impaired vascular tone, thrombosis, and inflammation. This imbalance is driven by oxidative and nitrosative stress, where ROS and RNS exceed antioxidant defenses. ROS/RNS can mediate both physiological and pathological processes depending on many circumstances, including their concentration. Key factors contributing to ED include mitochondrial dysfunction, uncoupling of eNOS, and dysregulation of NADPH oxidase enzymes, particularly NOX2 and NOX4, which play a role in endothelial activation and vascular injury. Aging exacerbates ED by increasing oxidative stress, mitochondrial damage, and inflammation, which further contribute to vascular remodeling, thrombosis, and the progression of CVDs such as hypertension and atherosclerosis. Reduced NO production and elevated ROS/RNS formation exacerbate endothelial damage, while changes in endothelial function, including altered EDHF signaling, increase the risk of CVDs and worsen vascular health. Addressing the ROS/RNS imbalance may not be sufficient, but it is critical to limit ED and its associated pathologies.

#### Impaired Antioxidant Defense

Vascular tissue contains several antioxidant systems, such as catalase, superoxide dismutase (SOD), glutathione peroxidase (GPx) and the thioredoxin (Trx)/selenoprotein thioredoxin reductase (TXNRD) [54,68,69]. Reduced activity of these systems leads to increased ROS production by oxidizing enzymes, which contributes to ED [68,69,70,71]. For instance, in endothelial cells, SOD1 inhibition elevated O_2_^•−^ levels and impaired fibroblast growth factor-2 and VEGF-induced extracellular signal–regulated kinases phosphorylation, thereby suppressing angiogenesis [71,72]. In animal models with SOD2 deficiency, vascular impairment has been observed in atherosclerosis and persistent pulmonary hypertension models [72,73,74]. Additionally, the lack of an antioxidative enzyme like GPx-1 accelerates and alters the progression of atherosclerotic lesions, particularly in ApoE^−/−^ mice, emphasizing the significant role of oxidative stress in the pathophysiology of atherosclerosis [60]. GPx is involved in both the conversion of H_2_O_2_ into water and molecular oxygen, as well as the reduction of lipid peroxides into alcohols [74]. GPx-1 deficiencies also have deleterious effects in atherosclerosis. Low levels of this enzyme are correlated with reduced bioactive NO, which is essential for maintaining vascular health. GPx-1 deficiency is directly related to the development of ED, inflammation and neointimal formation, suggesting a role of ROS in altering the amount and function of NO [74,75].

A beneficial role is ascribed to selenium; indeed, its intake directly regulates GPX1 expression and levels, with selenium supplementation reducing susceptibility to CVDs, while selenium deficiency increases the risk of CVDs [76,77]. GPX1 is a selenoprotein negatively affected by homocysteinemia, leading to oxidative stress, reduced NO bioavailability, and ED. Elevated homocysteine levels lead to reduced expression and activity of GPX1, resulting in increased ROS accumulation and endothelial damage. Conversely, GPX1 overexpression can restore vascular function, emphasizing its crucial role in mitigating hyperhomocysteinemia-induced dysfunction [78,79,80].

Furthermore, the Trx/TXNRD system is implicated in ED protection, linked to homocysteine-induced oxidative stress and atherogenesis. Reduced Trx activity, combined with compensatory TXNRD upregulation, strongly correlates with disease severity in coronary artery disease (CAD) patients under hyperhomocysteinemic conditions [81]. The oxidative stress caused by homocysteine-mediated downregulation of Trx1 likely drives TXNRD upregulation to counteract hydroperoxides and lipid peroxides associated with atherosclerosis [82]. Similarly, studies highlight NADPH oxidase activation and decreased Trx and eNOS activity as mechanisms linking high homocysteine levels to oxidative stress and ED [83]. A reciprocal regulation between GPX1 and TXNRD1 was observed during homocysteine-induced hypomethylation stress, driven by S-adenosylhomocysteine accumulation. This dysregulation promotes endothelial activation, inflammatory cytokine production, and a pro-atherogenic phenotype [84]. Recent findings demonstrate that selenium nanoparticles effectively alleviate ED and vascular inflammation in ApoE^−/−^ atherosclerosis model [85]. Furthermore, selenoproteins, particularly GPX1, play a pivotal role in preserving endothelial function, regulating vascular reactivity, promoting angiogenesis, and preventing adverse vascular remodeling. Their cardioprotective effects, especially in attenuating oxidative stress and endothelial dysfunction, are increasingly recognized [76,77].

Sodium-glucose cotransporter 2 (SGLT2) inhibitors are a class of drugs that act on SGLT2 transporters to decrease glucose reabsorption in the kidneys, thereby increasing glucose excretion in the urine and lowering serum glucose levels. SGLT2 inhibitors are emerging as drugs with multiple beneficial effects on various organs and pathological conditions, although their mechanisms of action are not yet fully understood [86,87,88,89]. Among the proposed mechanisms are the reduction of ROS levels, the enhancement of eNOS activity, and the increased availability of NO [89]. These last findings have been further confirmed by several authors, even in dysmetabolic mouse models. In fact, although SGLT2 is not the main glucose transporter in ECs, it has been observed that the reduction of oxidative stress by empagliflozin (EMPA), a selective inhibitor of SGLT2, improves the function of these cells, acting independently from its main pharmacological function [86,87,88,89]. The molecular pathway induced by EMPA involved Sestrin2/adenosine monophosphate-activated protein kinase (AMPK), increased phosphorylation of eNOS at Ser^1177^, and mechanistic target of rapamycin (mTOR) activation. In obese Sestrin2 knockout mice, these pathways are partially attenuated. The antioxidant action of EMPA seems to be mediated by Nrf2/HO-1, also suggesting an anti-inflammatory activity [89]. In apparent contrast to eNOS phosphorylation at Ser^1177^, this study found that EMPA inhibited Akt [89], which typically drives Ser^1177^ eNOS phosphorylation. Nevertheless, Sun et al. [89] observed AMPK activation, and since AMPK can also phosphorylate eNOS at Ser^1177^ [90,91], it is likely that, in the conditions considered by the authors [89], AMPK mediates Ser^1177^ eNOS phosphorylation.

The importance of abating redox stress is evidenced by the proposed treatment with antioxidant factors at multi-level intervention strategies, as extensively reviewed elsewhere [36,37,38,39,40,92]. One example is the observation that N-acetyl-L-cysteine (NAC) prevents these detrimental changes during calcific aortic valve stenosis in human valve endothelial cells, where oxidative stress and the accumulation of oxidation-induced protein S-glutathionylation drive a dysfunctional shift that promotes calcification.

*In summary*, vascular tissue contains antioxidant systems like SOD, catalase, GPx, and Trx/TXNRD, which protect against oxidative stress. Reduced activity of these systems leads to increased ROS/RNS production, contributing to ED and atherosclerosis. For example, SOD1 blockade increases basal O_2_^•−^ levels, and GPx-1 deficiency accelerates atherosclerotic lesion progression. Selenium intake regulates GPx-1 expression, reducing CVD risk, while homocysteine-induced oxidative stress reduces GPx-1 activity, worsening ED. The Trx/TXNRD system is also linked to ED protection, with its dysregulation in hyperhomocysteinemic conditions worsening oxidative stress and atherosclerosis. Selenium nanoparticles alleviate ED in atherosclerosis models. SGLT2 inhibitors, like EMPA, reduce oxidative stress and increase NO availability, improving endothelial function. Antioxidant treatments, such as NAC, also protect against oxidative stress-induced changes, preventing ED in many conditions, including calcific aortic valve stenosis.

### 3.2. Focus on Inflammation and Inflammasome

During infections and inflammatory processes, ECs undergo a mechanism known as endothelial activation, characterized by functional and morphological changes, as defined above. The responsible stimuli can be different, including inflammatory cytokines (TNF-α, IL and interferon-γ), bacterial endotoxins, or activation of the pattern recognition receptor (PRR) following the detection of pathogen-associated molecular patterns (PAMPs) or damage-associated molecular patterns (DAMPs) [93,94].

**Figure 2:**
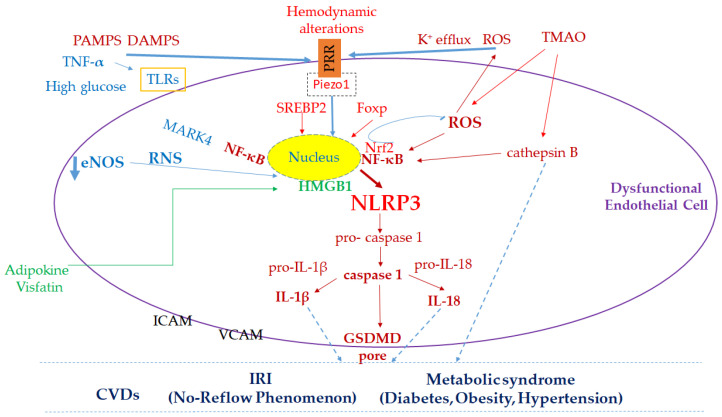
Key molecular mechanisms linking endothelial dysfunction, NLRP3 inflammasome, and cardiovascular diseases (CVDs). Schematic illustration of the pathways and factors contributing to endothelial dysfunction and the progression of CVDs. The activation of the NLRP3 inflammasome by PAMPs, DAMPs, and hemodynamic alterations triggers the production of inflammatory cytokines (e.g., IL-1β, IL-18) via caspase-1 activation and GSDMD pore formation. Key mediators such as ROS, RNS, high glucose, trimethylamine N-oxide (TMAO), and adipokines (i.e., visfatin), leading to NF-κB activation, further exacerbate the inflammatory response. Endothelial dysfunction is also associated with metabolic syndrome and ischemia/reperfusion injury (IRI), including the no-reflow phenomenon. The main pathways are highlighted in red; additional pathways (e.g., high glucose and eNOS downregulation) are shown in blue; and the pathway activated by adipokines is marked in green. Arrow colors correspond to their respective pathways. The protective pathway, Nrf2 activation, is included to illustrate hormesis and highlight its potential as a therapeutic target.

In addition to the aforementioned stimuli, other factors that can trigger PRR activation and therefore inflammasome formation include: K^+^ efflux, ROS, mechanical stimulation, and abnormal metabolites [94,95]. The action of ROS at the endothelial level is multifaceted. In fact, numerous co-factors are recognized, such as hyperglycemia, sarcoplasmic reticulum stress, senescence, and lipopolysaccharide (LPS) and IRI [92,95,96,97,98]. As depicted in Figure 2, many of the aforementioned factors can prime and activate the NLRP3 inflammasome. These processes are mediated by the upregulation of thioredoxin-interacting protein and nuclear factor kappa-light-chain enhancer of activated B cells (NF-κB), and modulation of nuclear factor erythroid-derived 2-like 2 (Nrf2) [98,99]. In particular, during myocardial damage, ischemic cardiomyocytes release ATP, which interacts with P2X7 receptors (P2X7R) to activate ECs, immune cells and potentially platelets [98,100,101,102], although the role of P2X7 in platelet aggregation and thrombosis remains unclear [103,104]. This receptor activation induces the opening of cation channels, initiating inflammatory processes [102,105,106]. Indeed, extracellular ATP can stimulate K^+^ efflux via P2X7R-gated K^+^ channels, contributing to the activation of the NLRP3 inflammasome [98,99,100,101,102,105,107]. Persistent stimulation of P2X7R results in the formation of nonselective membrane pores, allowing molecules up to 900 kDa to pass through. This ultimately leads to membrane disruption and apoptotic cell death [108]. P2X7R is expressed in ECs, predominantly localized on their apical membrane, and is implicated in ED under various stress conditions [94,98,100]. Indeed, K^+^ leakage is a common event in NLRP3 activation; the mechanism involves NIMA-related kinase 7 (NEK7), which directly binds to NLRP3, intervening in both its oligomerization and activation [99,102].

The triggered inflammatory response determines the activation of the NLRP3 inflammasome capable of promoting, with the cytokines produced, both oxidative stress and further inflammatory processes [7]. The formed NLRP3 inflammasome activates pro-caspase 1 to caspase 1, which in turn activates two cytokines, pro-IL-1β and inert pro-IL-18, to active IL-1β and IL-18, releasing them into the extracellular space [8] (Figure 2). In addition to NLRP3, NLRP1 and its absence in melanoma 2 have also been reported to be involved in the onset of ED [109]. The consequence of inflammasome activation is the triggering of secondary inflammatory mediators, such as IL-6 and C-reactive protein [110], leading to pyroptosis [111,112,113]. At the endothelial level, IL-1β leads to the secretion of adhesion molecules and chemokines, further increasing the inflammatory response. A vicious circle is therefore triggered that culminates in ED [98,112,113,114,115].

The action of caspase-1 is not limited to the activation of cytokines, but is also able to activate gasdermin D (GSDMD) resulting in membrane pore formation, cell death, and pyroptosis (Figure 2) [111,112]. Following activation by caspase-1, the GSDMD molecule undergoes oligomerization with the formation of pores at the membrane level, causing both cellular swelling and the release of intracellular components [98,116,117,118,119]. The released molecules amplify inflammation, acting as DAMPs. At the endothelial level, the action of the inflammasome not only causes endothelial dysfunction, which is the initial step, but cell death is also observed, causing the serious damage in the vascular system present in pathologies such as diabetic retinopathy and atherosclerosis, to name a few [109,113,114].

Mechanical stimuli, such as changes in blood flow, can activate NLRP3 in ECs, which are highly sensitive to hemodynamic variations (Figure 2). Indeed, flow alterations act as mechanosensory triggers, driving processes like vascular remodeling. These changes also induce increased NLRP3 expression in ECs via the activation of two key proteins: sterol regulatory element-binding protein 2 and forkhead box P (Foxp) [114]. In these conditions, the latter appears to be down-regulated following the reduction of Krüppel-like factor 2 (KLF2) [114]. In mouse lung ECs, mechanical stretching has been shown to activate NLRP3 and induce endothelial-to-mesenchymal transition (EndoMT), as further confirmed by studies using NLRP3 knockout mouse models [114,115].

Activation of the NLRP3 complex at the endothelial level may involve abnormal circulating molecules, such as TMAO, derived from dietary choline associated with atherosclerosis. This molecule determines NLRP3 priming/activation both by cathepsin B and oxidative stress (Figure 2) [116,117,118,119].

In the presence of high glucose levels, endothelial activation of the NLRP3 inflammasome has been observed, both in terms of cytokines IL-1β and IL-18, and related factors such as microtubule-affinity regulating kinase-4 (MARK4), toll-like receptors (TLRs), and/or increased nuclear translocation of the transcription factor NF-κB [94,119,120].

Recently, in a hypertensive rat model, it was observed that cystathionine γ-lyase (CSE) levels were significantly reduced while NLRP3, caspase-1, and related cytokines were elevated compared to the control model. This scenario was reversed by the exogenous administration of the hydrogen sulfide (H_2_S) donor sodium hydrosulfide (NaHS), which effectively mitigated endothelial damage by targeting NLRP3 activation and reducing oxidative stress. Similar protective data by NaSH were observed in human umbilical vein ECs pretreated with angiotensin II [121]. Moreover, cystathionine gamma-lyase (CGL) deficiency exacerbates isoprenaline-induced cardiac injury in mice, but this effect was mitigated by an H_2_S donor, which enhanced Sirtuin 1 (SIRT1) expression and its deacetylation activity through S-sulfhydration at zinc finger domains [121,122]. These findings suggest that reduced CGL production, secondary to oxidative stress in human aortic ECs and aorta from atherogenic ApoE^−^/^−^ mice, contributes to ED [123]. The protective action of H_2_S is not limited to the hypertensive model. In fact, its anti-inflammasome action has also been shown to be protective against ED. In particular, the protective action is carried out through the intervention of the peroxisome proliferator-activated receptor delta (PPARδ)/SGLT2/phospho-signal transducer and activator of transcription 3 (p-STAT3) pathway [124,125]. The interplay between CSE and the NLRP3 inflammasome can be considered as a yin/yang relationship, where these two elements have opposing but complementary roles in inflammation and cellular homeostasis: their dynamic interaction maintains a fine balance between adequate immune defense and the prevention of excessive, potentially harmful inflammation. The use of MCC950, a specific inhibitor of the NLRP3 inflammasome, reduces the release of the cytokines IL-1β and IL-18, following a cyclic stretch in pulmonary microvascular ECs [124]. Numerous other NLRP3 inhibitors have been explored as potential treatments for ED and CVDs [124,125,126] (See also Section 4.3); for comprehensive reviews, see [9,102,110,127].

*In summary*, ECs undergo activation during inflammation, influenced by cytokines, bacterial toxins, and PAMPs/DAMPs. Factors like ROS/RNS, mechanical stimuli, and abnormal metabolites also trigger NLRP3 inflammasome formation. ROS/RNS, hyperglycemia, and ischemia can prime the NLRP3 inflammasome, which produces cytokines such as IL-1β and IL-18, promoting ED. The activated inflammasome triggers cell death through pyroptosis, releasing ILs and DAMPs that further amplify inflammation. Mechanical stimuli, like blood flow changes, can also activate NLRP3 in ECs, contributing to vascular remodeling and endothelial-to-mesenchymal transition. Abnormal molecules like TMAO and high glucose levels also activate NLRP3, worsening endothelial damage. Besides NLRP3 inhibitors, other protective mechanisms, such as Nrf2 upregulation and H_2_S donors, can reduce NLRP3 activation and mitigate ED, highlighting a complex interplay between inflammatory processes and other cellular pathways contributing to ED.

## 4. Endothelial Dysfunction and Cardiovascular Diseases

Cardiovascular diseases are among the leading causes of mortality worldwide, with their development and progression closely tied to ED [9,33,38,86,92,98,110,127]. ED plays a critical role in the development of CVDs, metabolic syndrome, and related conditions. Aging exacerbates vascular dysfunction, leading to ED via oxidative stress and inflammation, which collectively accelerate the progression of CVDs. The following sections examine the dual role of ED in initiating and advancing CVDs. Moreover, we explore how preclinical aging models contribute to understanding the roles of oxidative stress, inflammation, and NLRP3 inflammasome activation in ED, aging and CVDs.

### 4.1. Ischemia Reperfusion Injury, Metabolic Syndrome, and NLRP3 Inflammasome

Myocardial IRI damages both cardiomyocytes and vessels. In brief, in cardiomyocytes, during ischemia, lack of O_2_ disrupts the mitochondrial respiratory chain, making ATP production inefficient. ATP synthase reverses, consuming ATP to pump protons, and low ATP levels inhibit the Na^+^/K^+^ ATPase, leading to Na^+^ accumulation and Ca^2+^ overload via the Na^+^/Ca^2+^ exchanger. This triggers the activation of lytic enzymes, causing cellular damage and death. Upon reperfusion, oxidative stress, reduced NO synthesis, and ATP resumption exacerbate injury, leading to hypercontracture, membrane rupture, and necrosis. pH restoration and Ca^2+^ overload induce mitochondrial permeability transition pores (mPTPs) opening, resulting in a loss of mitochondrial integrity, which plays a pivotal role in various cell death modalities for reviews see, [127,128,129,130,131,132,133].

Here, we focus on the pivotal role of the NLRP3 inflammasome in myocardial IRI by acting as a central mediator of the inflammatory response and tissue damage. Emerging evidence suggests that NLRP3 activation plays a crucial role in myocardial IRI [92,110,127,134,135,136,137]. Histological studies have shown the presence of NLRP3 accumulations in cardiomyocytes and endothelial cells within the ischemic region and border zones during the early stages of acute myocardial infarction. Subsequently, leukocyte infiltration and fibroblast activation occur. As inflammation resolves, NLRP3 spots become more prominent in cardiomyocytes and/or fibroblasts [135,138]. NLRP3 activation is triggered by mitochondrial stress, ROS generation, and the release of DAMPs, which amplify inflammation through caspase-1 activation and the subsequent release of pro-inflammatory cytokines, including IL-1β and IL-18 [92,110,127,134,135,136]. This process contributes to pyroptotic cell death, exacerbating endothelial and myocardial injury following ischemia [139]. Several studies have demonstrated that NLRP3 inhibition significantly mitigates I/R-induced damage by improving endothelial function and attenuating the inflammatory response [92,110,127,140,141,142,143].

The majority of the available evidence supports the notion that NLRP3 activation exacerbates myocardial IRI and highlights the therapeutic potential of targeting NLRP3 in the context of IRI. However, Sandanger et al. [144] observed a similar increased infarct size in both NLRP3^(−/−)^ and ASC^(−/−)^ mice. ASC (Apoptosis-associated Speck-like protein containing a CARD) is an adaptor protein that plays a crucial role in the activation of the inflammasome. This observation led the authors to propose a cardioprotective role for the NLRP3 inflammasome in myocardial IRI, concluding that targeting NLRP3 or ASC may not be beneficial in the context of acute myocardial infarction and revascularization therapy [144]. Nevertheless, as pointed out by Toldo et al. [145], this conclusion is not fully supported by the data, as troponin I levels—an indicator of myocardial damage—remained comparable between treated and control groups after 24 h of reperfusion, in Sandanger et al.’s study [144]. Consistent with a causative role of NLRP3 inflammasome in myocardial IRI, we demonstrated that mice subjected to a high-fat, high-fructose diet for 12 weeks exhibited increased NLRP3 expression alongside markers of oxidative metabolism, while simultaneously showing a downregulation of key components of the RISK (Reperfusion Injury Salvage Kinase) pathway. When these hearts were subjected to an ex vivo I/R protocol, they displayed larger infarcts and greater lactate dehydrogenase release compared to controls [146]. More recently, using INF200, a 1,3,4-oxadiazol-2-one NLRP3 inhibitor, we were able to efficiently reduce cardiac IRI and metabolic complications in an in vivo model of diet-induced metaflammation [126].

Indeed, a link between metabolic syndrome, ED, and NLRP3 inflammasome activation as major risk factors for IRI, increasing cardiovascular vulnerability to ischemic events, is established. In particular, obesity induces vascular dysfunction through chronic low-grade inflammation and excessive ROS production, leading to ED and reduced NO bioavailability [147]. Moreover, insulin resistance associated with metabolic syndrome impairs cellular signaling and compromises the ability of myocardium to adapt to ischemic stress [148]. Recent studies suggest that systemic inflammation and metabolic alterations in individuals with metabolic syndrome exacerbate IRI by intensifying the inflammatory response and promoting maladaptive tissue remodeling [126,149]. Therefore, targeted interventions aimed at modulating NLRP3 inflammasome and oxidative stress may offer promising strategies to mitigate ischemic damage in patients with metabolic syndrome.

#### Ischemia Reperfusion Injury and No-Reflow Phenomenon

Relevant to the concept of ED, it is important to note that IRI affects not only cardiomyocytes but also the coronary circulation, leading to a cascade of harmful events. These include the release of soluble factors from the damaged vessel, microembolization of debris, endothelial damage with increased permeability and edema, platelet activation, leukocyte adhesion, erythrocyte stasis, and a transition from vasodilation to vasoconstriction. These processes culminate in structural capillary damage, resulting in microvascular obstruction, intramyocardial hemorrhage, and the no-reflow phenomenon [127,150]. This phenomenon remains a major challenge, independently worsening prognosis regardless of infarct size. While advanced imaging techniques can now detect microvascular obstruction and intramyocardial hemorrhage with precision, effective therapeutic options for these complications are still lacking. In these conditions, the “no-reflow” occurs at the vascular level, where ED is occurring. According to Eeckhout and Kern, the no-reflow phenomenon is defined as “inadequate myocardial perfusion through a given segment of the coronary circulation without angiographic evidence of mechanical obstruction” [150].

Following ischemia and subsequent reperfusion, a reduced production of NO is observed, which characterizes ED, and a greater expression of adhesion molecules is recorded, which in turn facilitates the infiltration of leukocytes. The final result is the “no-reflow phenomenon”, accompanied by a lack of NO and an excess of RNS [127,150]. The result is vasoconstriction and formation of microthrombi, as well as activation of NF-κB, which is necessary for the expression/activation of NLRP3 (Figure 2). Indeed, oxidative stress also activates the vascular NLRP3 inflammasome, worsening endothelial dysfunction, the no-reflow phenomenon, and tissue damage during reperfusion [64,65,66].

From the inflammatory point of view, the role played by lymphocytes has been studied by several authors, who agreed that approximately 2 min after reperfusion an accumulation of T lymphocytes occurs. This was confirmed by using knockout mice devoid of mature lymphocytes, which showed reduced IRI. The damages were restored after the transfusion of mature lymphocytes into these animals [150,151,152]. Nevertheless, several in vitro and ex vivo studies, conducted in the absence of an immune component, have demonstrated that IRI, including no-reflow, and the associated cardioprotective protocols do not necessarily rely on cell-mediated immune mechanisms [153,154,155].

In a recent study by Lee et al. [156], it was observed that within the complex mechanism of no-reflow, other cell species come into play in addition to endothelial and immune. In an in vitro model, the authors identified *pericytes* as cells capable of contracting and releasing a vasoactive molecule in response to hypoxia and sympathetic stimuli, both of which are involved in the onset of no-reflow observed following IRI. Therefore, pericytes, which are particularly sensitive to hypoxia, are part of the vascular homeostasis, being located at the abluminal level of capillaries and microvessels, where they play an important role in hemodynamics by regulating blood flow and pressure at the level of microvessels and capillaries, both in physiological and pathophysiological states [156,157,158,159].

Endothelial dysfunction contributes to both the initiation and progression of myocardial IRI by impairing vasodilation, promoting inflammation, increasing oxidative stress, and facilitating pericyte contraction [156,157,158,159].

*In summary*, myocardial IRI can result in both myocardial damage and coronary ED, creating a vicious cycle that further aggravates myocardial injury. ED may involve processes such as neutrophil–endothelial interactions, platelet aggregation, vasoconstriction, and pericyte contraction, which play a significant role in the no-reflow phenomenon.

### 4.2. Metabolic Syndrome

Metabolic syndrome, characterized, among others, by insulin resistance, obesity, atherogenic dyslipidemia, and hypertension, is a major contributor to ED, a critical driver in the pathogenesis of CVDs. ED represents a pivotal link between metabolic disturbances and vascular damage, facilitating the progression of atherosclerosis and other cardiovascular complications. Among the systemic manifestations of metabolic syndrome, metabolic dysfunction-associated fatty liver disease (MAFLD) is particularly significant, as the hepatic counterpart of this syndrome. The rising global prevalence of MAFLD, closely tied to increasing rates of diabetes and obesity, underscores its relevance [160,161]. MAFLD encompasses a spectrum of liver abnormalities, ranging from steatosis to non-alcoholic steatohepatitis (NASH), with potential progression to cirrhosis and liver cancer. The complex interplay of pathophysiological mechanisms in MAFLD further exacerbates systemic metabolic and vascular dysfunction, highlighting its role in the broader context of CVD risk [161].

#### 4.2.1. Hyperglycemia and Diabetes

Insulin resistance, which causes alterations in lipid and glucose metabolism, results in hyperglycemia and hyperinsulinemia as the body tries to maintain normal glucose levels. These metabolic disturbances, along with obesity, atherogenic dyslipidemia, and hypertension, contribute to ED, which in turn exacerbates diabetes and cardiovascular risk [53,160,162]. At the endothelial level, hyperglycemia induces severe damage. In healthy subjects, glucose enters ECs via the glucose transporter 1 (GLUT-1) transporter regulated by extracellular glucose levels, whereas in diabetes, increased GLUT-1 activity leads to excessive intracellular glucose accumulation and advanced glycation end products (AGE) formation [163,164]. The rise in AGE levels leads to increased endothelial permeability, eNOS inhibition, and damage to DNA and proteins, leading to cellular injury [52,163]. However, hyperglycemia causes an increase in cellular proliferation following stimulation of growth factors (e.g., hepatocyte growth factor, VEGF). Hyperglycemia also induces pro-inflammatory factors [165] and expression of NLRP3 in ECs, increasing oxidative stress at mitochondrial level and apoptosis. The hyperglycemic condition is also characterized by a reduction in antioxidant enzymes (e.g., SOD 1 and 2) [53]. In these conditions, eNOS undergoes uncoupling with the production of O_2_^•−^ and consequent formation of ONOO^−^. The ROS and RNS produced reduce the action of BH4 and consequently the production of NO by ECs [53]. The alteration of eNOS and the action induced by AGEs induce inflammation, expression of growth factors, apoptosis, and increased permeability of ECs with a consequent increase in leukocyte adhesion that favor atherosclerotic plaque [166,167].

It has been suggested that, in diabetic subjects, ED is partly caused by alterations in red blood cells (RBCs), where both function and structure are affected by plasma LDL levels. Specifically, a high LDL concentration has been associated with reduced deformability and increased rigidity of RBCs [168]. Altered RBCs contribute to the disruption of purinergic signaling observed in ED, exacerbating oxidative stress through a reduction in miR-210 levels and an increase in peroxynitrite-dependent arginase activity, thereby further promoting ED [169].

It is worth noting that hyperglycemia and insulin resistance lead to hyperactivation of the renin-angiotensin-aldosterone system (RAAS), contributing to ED, vascular and cardiac fibrosis, VSMC proliferation, and remodeling, ultimately resulting in arterial stiffness [169]. Clinically, patients with metabolic syndrome exhibit increased pulse wave velocity due to this arterial stiffness. This increase in pulse velocity may alter hemodynamic, thus exacerbating ED. The development of heart failure with preserved ejection fraction (HFpEF) in metabolic syndrome is closely linked to ED. Metabolic syndrome, including diabetes, dyslipidemia, and hypertension, consists of classical risk factors and comorbidities for both ED and HFpEF [170].

#### 4.2.2. Obesity

Obesity, a key component of metabolic syndrome, is associated with chronic low-grade inflammation that disrupts vascular homeostasis, contributing to ED. Under normal conditions, healthy perivascular adipose tissue (PVAT) promotes blood vessel dilation. However, in obesity, alterations in the adipocytokines released by PVAT lead to reduced vasorelaxation, increased oxidative stress, and upregulation of pro-inflammatory cytokines and nicotinamide adenine dinucleotide phosphate (NADP) oxidase. These changes also trigger macrophages in PVAT to transform into pro-atherogenic phenotypes, driven by macrophage-derived exosomes induced by oxLDL, further exacerbating ED [41]. The NLRP3 inflammasome is central to obesity-induced inflammation and ED [41]. Activated by adipocyte-derived factors like *visfatin*, it stimulates caspase-1, promoting the release of IL-1β and IL-18, which drive endothelial inflammation [171]. In obesity, NLRP3 detects danger signals, contributing to insulin resistance and glucose dysregulation through adipose tissue inflammation and a pro-inflammatory T-cell shift [172,173,174]. High-fat diets amplify endothelial damage by activating NLRP3, disrupting vascular junction proteins, and increasing high-mobility group protein 1 (HMGB1) expression (Figure 2) [174,175].

#### 4.2.3. Hypertension

Hypertension, another important feature of metabolic syndrome, is a complex condition, affecting approximately 35–40% of the world’s adult population [176]. Hypertension is a risk factor for a number of diseases, such as stroke, heart failure, myocardial infarction, chronic kidney disease, and vascular dementia, and, in the most severe cases, premature death [177]. In the coming years, it is estimated that the number of adults affected by hypertension globally will increase. They were 1 billion in 2000 and will be 1.5 billion in 2025 [178].

Endothelial dysfunction is both a cause and an effect of hypertension. NO deficiency and ROS overproduction at the endothelial level are among the many molecular factors implicated in the development of hypertension. In fact, population studies have shown that oxidative stress markers are increased in patients with hypertension, and increased oxidative stress has been reported to be a risk factor for the onset of hypertension in subjects with normal blood pressure levels [179,180,181]. Indeed, it seems that excess ROS produced in hypertension is responsible for several features of the disease, including ED itself, as well as vascular damage, vascular hyperreactivity, arterial remodeling, renal/glomerular damage, increased activation of the sympathetic nervous system, activation of immune cells, and inflammation [181]. Furthermore, in hypertension, ED is associated with a deficiency of NO due to excessive production of O_2_^•−^, which leads to the production of ONOO^−^ [157]. As said, ONOO^−^ in turn leads to the oxidation of BH4, a NOS cofactor, with the formation of dihydrobiopterin (BH2) [181,182,183,184]. This molecule, BH2, competes with BH4 at the level of eNOS, promoting its uncoupling. The uncoupled eNOS transfers an electron from NADPH to O_2_, producing more O_2_^•−^ and worsening vascular dysfunction [184,185]. It is important to note that this mechanism of BH4 oxidation has been observed in the clinical setting [177].

Causes of eNOS uncoupling include S-glutathionylation, a reversible oxidative post-translational modification [185,186]. This post-translational modification plays an important role in ED and is associated with angiotensin II-induced hypertension in murine models [186,187]. The uncoupled eNOS is degraded through heat shock cognate 70 chaperone-mediated autophagy, followed by its transport to a lysosome-associated membrane protein-2A vesicle, where it is broken down by lysosomal proteases [187,188]. Degradation of uncoupled eNOS reduces oxidative stress; however, it simultaneously decreases NO availability, thereby sustaining ED and hypertension [187,188,189].

The produced ROS leads to activation of the NLRP3 inflammasome, resulting in activation of caspase-1, cleavage of pro-IL-1β and pro-IL-18, and the production of active forms of IL-1β and IL-18, which increase the inflammatory response, fibrosis, and vascular remodeling in hypertension [181,182]. Emerging evidence highlights ED induced by NLRP3 inflammasome activation as a critical factor in hypertension development. Low-grade inflammation, driven by both the innate and adaptive immune systems under the “danger model” plays a central role. The NLRP3 inflammasome acts as a pivotal signaling platform, contributing to endothelial damage and dysfunction in hypertension-associated conditions, including vascular smooth muscle remodeling and proliferation [188,189].

Recent studies have highlighted an involvement of the microbiota in hypertension, as demonstrated by data on its composition in hypertensive patients compared to healthy subjects [189,190,191,192,193]. The metabolite produced at the level of the intestinal microbiota involved in CVDs, including hypertension, through an oxidative stress mechanism, is TMAO [192,193]. TMAO derives from the metabolism of l-carnitine and choline present in red meat and other food sources of animal origin. A study conducted of over 5000 subjects (aged > 65 years) has allowed us to establish that a higher meat consumption can be associated with a higher incidence of CVDs and this association is partly linked to metabolites related to TMAO [193]. TMAO causes increased production of ROS at the mitochondrial level, inhibition of SOD2 and SIRT3 in ECs with consequent ED and inflammatory state following activation of the NLPR3 inflammasome (Figure 2) [194]. TMAO action is also recognized in ED due to aging, in which increased production of ROS, uncoupling of eNOS, and consequent reduced availability of NO are observed [195,196].

The action of antioxidant enzymes can determine deglutathionylation, restoring the NOS activity and therefore the production of NO. Among the antioxidant enzymes in this activity, Trx is worth mentioning; in fact, its overexpression restores eNOS function, improving endothelial function and hypertension in mouse models [196,197]. Treatment with SOD has resulted in reductions in blood pressure, ED, cardiovascular remodeling, and renal damage in experimental models [196,197,198]. It has been observed that eNOS can undergo uncoupling following the impairment of its eNOS dimerization due to the oxidation of cysteine residues. Cysteine oxidation interrupts the zinc-sulfur complex in the eNOS dimer and, consequently, the synthesis of NO, with consequent production of O_2_^•−^ [198,199]. Despite this, several large clinical studies [178,200,201] have reported that treatment with antioxidants does not lead to any improvement in hypertension and does not reduce cardiovascular events compared to placebo. The precise causes are not entirely clear; probably, multiple factors contribute, including the type of antioxidants chosen and their dosage and duration of treatment, or the patient selection criteria used. For example, most studies included patients with severe CVDs, and as a result, treatment with antioxidants was ineffective, further supporting the idea that these conditions require a multi-target approach.

*In summary*, metabolic syndrome, characterized, among others, by insulin resistance, obesity, and hypertension, is often accompanied by ED. Hyperglycemia, resulting from insulin resistance, induces severe endothelial damage via increased GLUT-1 activity and the accumulation of AGEs, leading to oxidative stress, inflammation, and apoptosis, characterizing diabetes. Insulin resistance also hyperactivates the RAAS, contributing to vascular fibrosis and arterial stiffness, increasing the risk of HFpEF. Obesity exacerbates ED through chronic inflammation and changes in perivascular adipose tissue, while hypertension causes ED via excessive ROS production and eNOS uncoupling, contributing to endothelial damage. Endothelia dysfunction linked to NLRP3 inflammasome activation is central to metabolic syndrome and hypertension, driven by low-grade inflammation involving the innate and adaptive immune systems. NLRP3 contributes to vascular smooth muscle remodeling and proliferation, exacerbating hypertensive damage. The relationship between oxidative stress, inflammation, and ED is a key factor in the progression of CVDs associated with metabolic syndrome. Antioxidants have shown limited efficacy in hypertension treatment, with recent studies focusing on microbiota-related metabolites like TMAO, which enhances ROS production and worsens endothelial function. This suggests that ED is multifactorial in nature.

### 4.3. Cardiovascular Aging and Endothelial Dysfunction

As previously stated, the aging process significantly impacts vascular function, contributing to ED, oxidative stress, and inflammation, all of which accelerate the progression of CVDs. Various preclinical models have been employed to study cardiovascular aging, and the mechanisms underlying these changes, providing valuable insights into potential therapeutic strategies. Cardiovascular aging is characterized by progressive vascular dysfunction, including arterial stiffening, ED, and impaired NO signaling, which contribute to increased cardiovascular risk. This paragraph summarizes key findings from aged animal models and their implications for cardiovascular health.

The Fischer 344 (F344) rat, a well-established model of aging, has been instrumental in elucidating mechanisms of vascular stiffness. Studies in aged F344 rats demonstrated that blockade of CD47, a cell surface receptor, can reverse coronary microvascular dysfunction [202]. Additionally, parathyroid hormone (PTH) has been shown to improve endothelial function in this model by enhancing eNOS expression, thereby promoting NO-mediated vasodilation [203]. These findings underscore the potential of targeting molecular pathways involved in NO signaling to combat vascular aging.

Similarly, the SAMP8 mouse, another aging model, exhibits pronounced vascular dysfunction. In this model, inflammation in perivascular adipose tissue is implicated in vascular dysfunction by causing the disappearance of an anticontractile effect. Melatonin treatment has been associated with increased expression of vasoactive factors, which improve endothelial function and overall vascular health [204]. This highlights the role of antioxidant and anti-inflammatory agents in mitigating the effects of vascular aging.

The C57BL/6 mouse, including its aged and high-fat diet (HFD)-induced cardio-metabolic syndrome variants, has provided further insights into vascular aging. Nitrate supplementation in aged C57BL/6 mice improved vascular function by influencing NO downstream signaling. Nitrite treatment has also proven effective in reversing endothelial dysfunction through increased NO bioavailability [205,206]. Similarly, short-term sodium nitrite therapy reversed vascular dysfunction and reduced large elastic artery stiffening [207,208].

Also, in the aged Sprague Dawley rat model, dietary nitrate supplementation has been shown to restore endogenous NO generation through the nitrate-nitrite-NO pathway [209]. This pathway is essential for maintaining vascular tone and endothelial function. Collectively, these studies suggest that dietary nitrates and nitrites could serve as practical and valuable therapeutic tools for addressing age-related cardiovascular dysfunction.

In a model of aged mice, EMPA also enhanced endothelial function and decreased arterial stiffness, suggesting that SGLT2 inhibition could be a promising therapeutic strategy to mitigate the progression of cardiovascular diseases in older adults [210]. Additionally, oral supplementation with apigenin, a flavonoid, has shown promise in aged C57BL/6 mice. Apigenin enhanced NO bioavailability, reversed age-associated aortic wall stiffening, and suppressed vascular inflammation, indicating its potential as a natural, dietary intervention for vascular aging [211]. Additional insights into interventions for vascular aging derive from aged Wistar rats. In this model, oleuropein supplementation, derived from olive leaves, has been found to prevent endothelial dysfunction and reduce macrovascular inflammation [212]. Furthermore, nitrate supplementation in this model improves vascular function, reinforcing the importance of dietary approaches to enhance NO signaling and vascular integrity.

The aged B6D2F1 mouse model is widely utilized to investigate oxidative stress-mediated vascular dysfunction. Research indicates that caloric restriction preserves NO bioavailability, thereby improving arterial function and mitigating age-related vascular impairments. Additionally, the SIRT1 activator SRT1720 has demonstrated efficacy in reversing endothelial dysfunction and vascular inflammation in a mice model, highlighting the critical role of the SIRT1-eNOS axis in vascular health [213,214,215]. Additionally, the Sprague Dawley rat model, when combined with a high-fat diet (HFD), mimics metabolic syndrome, a condition closely linked to vascular dysfunction. In this context, the use of Angelica gigas extract (a popular traditional medicinal herb widely used to treat dyslipidemia, owing to its antioxidant activity) has been shown to reduce atherosclerotic plaque formation and enhance NO bioavailability via the SIRT1-eNOS signaling axis [216].

Intriguingly, the role of physical activity and NLRP3 inflammasome have also been emphasized in an aging model. Exercise was shown to attenuate NLRP3 activation in obese mice coronary arterioles, preserving eNOS expression and NO bioavailability. This supports the notion that lifestyle modifications, such as increased physical activity, can significantly counteract vascular aging [217]. Moreover, using the atherosclerosis model ApoE^−/−^ mouse, the NLRP3 inflammasome emerges as a central target in the fight against ED and CVDs, as evidenced by recent studies highlighting various therapeutic strategies. For comprehensive reviews on the role of the NLRP3 inflammasome in ED and CVDs, see [9,102,110,127]. For example, in ApoE^−/−^ mice, oridonin and tacrolimus have been shown to attenuate atherosclerotic progression by inhibiting NLRP3 activation [218,219], while agents like sodium tanshinone IIA sulfonate, idebenone combined with rosuvastatin, and metformin exhibit additional benefits by modulating oxidative stress or associated signaling pathways [220,221,222]. Innovative compounds such as paeonol, peperomin E, and VX765 further illustrate the potential of targeting NLRP3 in mitigating inflammation and improving plaque stability [113,223,224,225]. Notably, lifestyle interventions like aerobic exercise and dietary supplements, including vitamin D, quercetin, and baicalin, also reduce vascular inflammation via NLRP3 pathways [217,226,227,228,229], emphasizing the importance of integrative approaches in managing this condition.

*In summary*, these preclinical models shed light on key mechanisms, including harmful processes such as oxidative stress, inflammation, and NLRP3 inflammasome activation, as well as protective pathways like the nitrate-nitrite-NO cascade and the SIRT1-eNOS axis. They offer valuable insights for developing strategies to preserve vascular health and mitigate age-related cardiovascular risks. The findings underscore the therapeutic potential of exercise and metabolic strategies, and molecular interventions, such as targeting NO signaling and reducing oxidative stress, in combating cardiovascular aging and dysfunction. Ongoing research with diverse models, including larger animals, will be essential for translating these discoveries into effective therapies for humans.

## 5. Conclusions

A critical factor in the pathogenesis of CVDs is the ED, which alters the delicate balance between vasodilation and vasoconstriction, ultimately resulting in impaired vascular tone, flow disruption, inflammation, and thrombosis. The primary contributors to ED include reduced NO production, increased oxidative/nitrosative stress, and the activation of pro-inflammatory pathways. These factors, alongside the aging process and altered antioxidant defenses, accelerate vascular damage and increase susceptibility to CVDs, particularly in conditions such as metabolic syndrome and atherosclerosis. The complex interplay between oxidative/nitrosative stress, inflammation, and ED highlights the importance of understanding the underlying mechanisms driving these processes. While antioxidant treatments have shown some promise in alleviating ED, their efficacy remains limited, indicating the need for alternative therapeutic strategies. Recent advances in targeting the NLRP3 inflammasome offer new potential avenues for treating ED and associated vascular diseases. The exploration of microbiota-related metabolites, like TMAO, and their role in exacerbating endothelial injury opens new perspectives for therapeutic intervention. Additionally, interventions that regulate metabolic disturbances, like those seen in insulin resistance and obesity, may help mitigate the progression of ED and related cardiovascular complications. Future research should focus on the development of targeted therapies that address multiple pathways, including dysmetabolic, inflammatory, and oxidative stress pathways involved in ED. Of course, implementation of preventive measures for modifiable risk factors, such as smoking cessation—including the use of electronic and heated tobacco cigarettes, which pose significant risks to cardiovascular health—is essential [6].

## Figures and Tables

**Table 1:** Factors Involved in Maintaining Endothelial Physiological Functions.

Substance	Precursor Compound	Effect	Other Effects	Secretion	Refs.
NO	L-arginine	Vasodilatation	Maintains basal tone of vessels; inhibits PLT activation, adhesion secretion and aggregation; promotes platelet disaggregation; inhibits leukocyte adhesion; inhibits smooth muscle cell migration and proliferation	Paracrine/Constitutive and induced by thrombin, ADP, BK, substance P, muscarinic agonists, shear stress, cyclic strain, cytokines	[11,15]
PGI_2_	AA	Vaso-dilatation	Delays platelet aggregation and deposition	Paracrine/Induced at sites of vascular perturbation	[14,16]
PAF	AA	Vasoconstriction	Favors leukocyte adhesion at cell surface	Juxtacrine/Induced by hypoxia and ischemia	[11,17]
ETs	Prepro-ET-1	Vasoconstriction	Mitogen for VSMCs; modulates effect of numerous compounds	Paracrine/Induced by shear stress, hypoxia, and ischemia	[18,23]
TxA2	AA	Vasoconstriction	Platelet aggregation	Typically produced by PLTs, but also byby ECs	[11,19]
Potassium ions, H_2_O_2_, lipid mediators (EETs)	Endothelial-derived molecule, AA	Vaso-dilation via hyper-polarization	Modulation of vascular tone, involvement in vasomotion	Secreted by ECs, following various stimuli	[14,20]
Calcium ions (Ca^2+^)	Mobilized intra-cellularly	Activates NO and EDH signaling	Facilitates negative feedback during vasoconstriction	Transferred between ECs and VSMCs via MEJs	[14,21]
Electrical signals	Hyperpolarizing current (via MEJs)	Hyper-polarization of VSMCs	Enables bidirectional signaling, supports myoendothelial feedback	Conducted through MEJs	[14,22]

AA: Arachidonic Acid; BK: Bradykinin; EETs: Epoxyeicosatrienoic Acids; PAF: Platelet Activating Factor; PLTs: Platelets. Other acronyms as in the text.

## Data Availability

No new data were created for this review article.
